# Letter in Response to Doyen et al., “Early Toxicities After High Dose Rate Proton Therapy in Cancer Treatments”

**DOI:** 10.3389/fonc.2021.687593

**Published:** 2021-05-13

**Authors:** Pierre Montay-Gruel, Marie-Catherine Vozenin, Charles L. Limoli

**Affiliations:** ^1^ Department of Radiation Oncology, University of California, Irvine, Irvine, CA, United States; ^2^ Laboratory of Radiation Oncology, Department of Radiation Oncology, Lausanne University Hospital and University of Lausanne, Lausanne, Switzerland

**Keywords:** proton, flash, lung cancer, normal tissue toxicity, high dose rate

Ultra-high dose rate “FLASH” radiation therapy (FLASH-RT) has attracted considerable attention if not excitement in the radiation oncology field. This enthusiasm has been based on the growing evidence showing that FLASH-RT prevents the development of the majority of normal tissue complications associated with radiotherapy while maintaining isoefficient tumor control, an effect defined *in vivo* and termed the “FLASH effect”. In their recent retrospective study, Doyen et al. (1) investigated the normal tissue toxicities developed by 127 cancer patients treated by “high dose rate” proton therapy. They reported that proton therapy delivered at a mean dose rate of 10 Gy/s triggered significant acute and subacute toxicities on different organs including the skin, eye, GI and brain. While the authors express their surprise and concerns about this absence of normal tissue protection, a careful examination of the published literature would have revealed that these results were in fact not unexpected since the reported studies did not implement dose rates remotely close to those required to achieve a FLASH effect.

The first report of the FLASH effect observed with a pulsed electron beam was performed at a mean dose rate above 40 Gy/s (2). Later on, Montay-Gruel et al. (3) assessed the dose rate dependence of normal brain toxicity with a dose rate escalation study and showed that cognitive function was fully preserved above 100 Gy/s, with a significant threshold of 40 Gy/s, above which FLASH-irradiated animals performed significantly better than the conventional dose rate group. More importantly, no normal tissue preservation was observed for dose rates around 10 Gy/s, similar to what was described by Doyen et al. (1). Thus, results reported by Doyen et. al (1), are confirmatory rather than surprising and document the expected normal tissue toxicities resulting from irradiation procedures that have been in the literature for decades. Concerning preclinical results observed with “true” ultra-high dose rate protons, normal tissue protection was observed in the mouse gut, skin and muscle for mean dose rates between 50 and 120 Gy/s (4–6), all well above the threshold of 40 Gy/s.

Notwithstanding, other studies have reported negative or uncertain “high-dose rate” data observed with electrons (7) or protons (8). In this regard, several groups have recently highlighted the importance of recording and reporting the radiation beam parameters used in studies claiming to deliver the requisite high-dose rates necessary to obtain an *in vivo* FLASH effect (9–11). The intent here was to enhance scientific rigor and reproducibility by clearly defining critical beam parameters including mean and instantaneous dose rate, beam on time, dose per pulse, total dose and pulse repetition frequency (12, 13). Given the parameters reported (and not reported) in the study by Doyen et al. (1), it is highly unlikely that their proton beam was within the parameters necessary to achieve a FLASH effect.

In conclusion, while the description of negative results in the investigation of the FLASH effect is essential to identify the limitations of FLASH-RT, the misleading use of “high dose rate” which is clearly a relative term, must be placed in the proper context so that the field of radiation oncology can properly interpret experimental results. Logical extension of these arguments leads to an alternative conclusion that nothing in the study published by Doyen et al. (1), was in fact surprising, as their dose rates were far from those required to ever achieve a FLASH effect.

## Author Contributions

PM-G, M-CV, and CLL wrote the letter. All authors contributed to the article and approved the submitted version.

## Funding

This work was funded by NIH program project grant PO1CA244091.

## Conflict of Interest

The authors declare that the research was conducted in the absence of any commercial or financial relationships that could be construed as a potential conflict of interest.

The reviewer CS declared a past co-authorship with one of the authors CL to the handling editor.
